# The Influence of Mental Health Literacy, Migration, and Education on the Duration of Untreated Psychosis

**DOI:** 10.3389/fpubh.2021.705397

**Published:** 2021-07-23

**Authors:** Naomi Takizawa, Ingrid Melle, Elizabeth Ann Barrett, Mari Nerhus, Akiah A. Ottesen

**Affiliations:** ^1^Department of Community Medicine, Faculty of Medicine, Institute of Health and Society, University of Oslo, Oslo, Norway; ^2^NORMENT; Division of Mental Health and Addiction, Institute of Clinical Medicine, University of Oslo, and Oslo University Hospital, Oslo, Norway; ^3^Early Intervention in Psychosis Advisory Unit for South East Norway (TIPS Sør-Øst), Division of Mental Health and Addiction, Oslo University Hospital Trust, Oslo, Norway; ^4^Division of Mental Health, Department for Specialized Psychiatry, Akershus University Hospital, Lørenskog Municipality, Norway

**Keywords:** early intervention, education, immigrants, mental health literacy, schizophrenia spectrum and other psychotic disorders, time-to-treatment, duration of untreated psychosis

## Abstract

**Background:** Duration of untreated psychosis (DUP) is associated with outcome in psychotic disorders and influenced by contextual factors such as immigration. Here we aimed to investigate the effect of mental health literacy (MHL) on duration of untreated psychosis considering the influence of migration and education.

**Methods:** A total of 269 participants who received their first adequate medical treatment for a psychotic disorder within the current or past year were included to the Thematically Organized Psychosis study in Oslo, Norway. Sociodemographic and clinical information was collected through systematic interviews. MHL was measured as “recognition of psychotic symptoms” and assessed by “The Attitudes and Beliefs about Mental Health Problems” schizophrenia version. Influence of education, migration and MHL on DUP was analyzed with hierarchical block-wise multiple regression analysis.

**Results:** Recognition of psychotic symptoms explained a small but unique variance (2.3%) in DUP after the effects of other important predictors were controlled for. Longer DUP was also associated with less education, lower premorbid social, and academic functioning, a diagnosis within schizophrenia spectrum disorder, and earlier age of onset. The model explained 26% of variance in DUP. Migration after the age of six and length of education were associated with MHL but did not have a significant interaction with MHL in predicting DUP.

**Conclusions:** MHL, measured as recognition of psychotic symptoms, has a small but significant independent effect on DUP. The effect of MHL was larger than years of education and migration history, and did not interact with either, in predicting DUP. This suggests that MHL is an independent factor in prevention strategies for early psychosis.

## Introduction

Developing a psychotic disorder may have long-lasting consequences, in terms of reduced productivity, psychosocial distress (1), and financial burden (2). Studies have shown that early treatment, and a shorter *duration of untreated psychosis* (DUP) is associated with better outcome of psychotic disorders (3, 4).

First generation immigrants are shown to have longer DUP (5), and more specifically those who have migrated after the age of compulsory school start (6). First generation immigrants with psychosis have more treatment delay than other groups (7). These findings are of interest as immigrants are at heightened risk of psychosis (8, 9). Moreover, people among ethnical minority groups are reported to be less likely to utilize mental health services (10, 11).

There could be several reasons for the longer DUP in first generation immigrants. DUP could be related to cultural background or education levels. However, it has been reported that health literacy is a stronger predictor of a person's health than other socioeconomic status such as education and ethnicity (12–14). We do not know if this is also the case for *mental* health literacy. The positive effect of increased knowledge about mental health on DUP has been indicated by the Treatment and Intervention in Psychosis (TIPS) study from Norway, which showed information campaigns, when combined with an early detection service, resulted in shortening average DUP from 16 to 5 weeks (15). These findings suggest that increased knowledge about psychosis in the general public is beneficial in reducing DUP.

Knowledge about psychosis is an important aspect of *mental health literacy* (MHL) defined as “knowledge and beliefs about mental disorders which aid their recognition, management or prevention” (16). In general, MHL increases help-seeking behavior (17, 18), but there seems to be cross-cultural and cross-national differences with greater MHL in developed countries compared to developing countries (19). We have previously found that beliefs about psychosis measured with a MHL instrument differ between ethnic groups in their first episode of psychosis even though there were no significant differences in insight into one's own condition and treatment (20). Still, we do not know if MHL in immigrants influence DUP.

In this study we thus aim to explore further the role of MHL in predicting DUP for persons in their first episode of psychosis, with a specific focus on the role of migration, as well as education. Efforts to understand this can generate knowledge which may help design interventions for shortening DUP in high-risk populations such as immigrant groups.

## Materials and Methods

This study used data collected for the Thematically Organized Psychosis (TOP) study in Oslo, Norway. The study was approved by the Regional Committee for Medical Research Ethics (ref # 2009/2484). Our research methodology conformed to the Code of Ethics of the World Medical Association, Helsinki Declaration. Participants were recruited from 2006 to 2010, from both in- and outpatient units in five major hospitals in the South Eastern region of Norway. The study had a cross-sectional design including a non-selected consecutively recruited catchment area sample of patients with a DSM-IV psychotic disorder.

### Sample

Clinicians from the recruitment units were asked and reminded at regular intervals to refer all patients with a clear or potential diagnosis of any psychotic disorder to the study. The treatment units served all patients living in these areas and there were no alternative psychiatric services offering treatment for psychotic disorders, thus reducing possible recruitment bias.

Participants were diagnosed according to the Diagnostic and Statistical Manual of Mental Disorders, Fourth Edition (DSM-IV) and the diagnostic distributions was: schizophrenia spectrum disorders including schizophrenia, schizoaffective disorder, schizophreniform disorder, (*n* = 137, 51%), other psychotic disorders (*n* = 73, 27%), and bipolar disorders (first adequate treatment for a manic episode within the current or past year) (*n* = 59, 22%). Participants who had IQ measures below 70, and/or inability to speak a Scandinavian language were excluded. We also excluded cases where the psychosis was assessed as being substance induced or clearly associated with a somatic or organic condition.

Information on immigrant background was collected through participant interviews where we asked for the country of birth for the participant's parents, themselves, and migration history. We categorized the information according to definitions used by the national bureau of official statistics “Statistics Norway” at the time of inclusion (21) which are; foreign born of foreign-born parentage (first-generation immigrants), Norwegian born to foreign born parent(s) (second-generation immigrants), and other immigrants which included persons born to one Norwegian parent in or outside of Norway.

### Assessments

All participants were assessed by trained clinical research personnel, either medical doctors, or clinical psychologists. The Norwegian version of The Structured Clinical Interview for DSM-IV (SCID-I) was used for diagnostic purposes. As there exists some discrepancy in the definition of adequate treatment in the operationalization of DUP (22) we used the same definition as other research studies in a Norwegian context (3). DUP was measured as weeks from the first verified psychotic symptom [defined as a score over clinical cut-off (≥4, on a Likert scale from 1 to 7) on the items P1—delusions, P3—hallucinatory behavior, P5—grandiosity, P6—suspiciousness/persecution, or G9—unusual thought content on the Positive and Negative Syndrome Scale (PANSS) (23)] to when adequate treatment for psychosis was initiated (i.e., admission to hospital for psychosis or receiving antipsychotic medication).

Mental health literacy was assessed with the self-report questionnaire “Attitudes and Beliefs about Mental Health Problems, schizophrenia version” (24) which was developed specifically for this purpose. As a part of the TOP study, the questionnaire was translated into Norwegian, back translated into English and approved by the original author. Participants were asked to read a vignette describing a person with symptoms of schizophrenia according to the ICD-10 (25) and DSM IV (26), and then answer question about their personal assessment of the persons condition and symptoms, causes and treatment alternatives. To assess recognition of psychotic symptoms we used the answers from the open-ended question: “With the information given, if anything, what is wrong with Ola (the person described?).” The responses were then categorized in accordance to previous published studies (20, 27) as “1—psychosis,” “2—mental disorder” or “3—other explanations.” The category “psychosis” was chosen when participants mentioned one or more of the following: “psychosis,” “schizophrenia,” “delusions,” “paranoid,” or “hallucinations.” The category “mental disorder” included depression, anxiety, obsession, or compulsion. Responses other than these were categorized as “Other explanations” such as concrete answers, situational explanations, or statements of uncertainty.

Premorbid function was assessed by the Premorbid Adjustment Scale (PAS) (28). The participants were asked about social and academic aspects of their life from childhood, through early and late adolescents and adulthood up until 6 months before the onset of psychotic symptoms (29). Scores were assessed by the interviewer on a 6-point Likert scale (0–6) with higher scores representing lower premorbid adjustment. In this study, PAS scores were split into academic and social scores as used in a previous study (6). “PAS social” and “PAS academic” represent participants' social and academic function levels of the latest life period before illness onset, respectively.

### Statistical Analyses

The analyses were performed using Statistical Package for the Social Sciences (SPSS) for Windows, version 26.0 (IBM Corp., Armonk, USA, 2017). The level of significance was pre-set to *p* < 0.05 (two tailed). Analysis of variance (ANOVA) and Chi-square test were used to compare demographic and clinical variables between groups, and the non-parametric test Kruskal-Wallis when applicable. For *post-hoc* analysis we applied Bonferroni correction for continual variables and for categorical variables we used standardized residuals interpreting those above 2.00 as contributing significantly. DUP is highly skewed and therefore log transformed for the between group comparison of this variable.

Hierarchical multiple regression analyses were used to investigate predictors of DUP. Because of the skewed distribution of PAS social, PAS academic, total years of education, and age of onset as well as DUP these variables were logarithmically transformed for the regression analyses. The model includes interaction terms, and the goal was to compare effects of different predictors on the outcome (DUP). Therefore, to reduce multicollinearity, and find the model with best fit we calculated the mean and standard deviation of the variables in the model on a standardized scale. Further, the Pearsons correlations matrix indicated high correlation between age and age of onset (*r* = 0.79), and Caucasian/Non-Caucasian and migration after the age of six (*r* = −0.49) (**Table 2**), and we therefore only included age of onset and migration after the age of six in the regression analyses.

The final model included gender (block 1), PAS social and PAS academic (block 2), age of onset and diagnosis of schizophrenia spectrum disorder (block 3), migration after the age of six (block 4), total years of education (block 5) and recognition of psychotic symptoms in the final block (block 6). These variables were chosen through careful consideration of previous studies (6, 30) as well as the associated variables in line with the hypothesis of the study. Possible interaction between recognition of psychotic symptoms, migration after the age of six and total years of education were analyzed in a two-way analysis of variance (ANOVA) and in a regression model. The data that support the findings of this study are available from the corresponding author upon reasonable request.

## Results

### Sociodemographic Characteristics

A total of 269 participants in their first episode of psychosis were included. The sample consisted of 60% males and the mean age was 27 (See Table 1 for sample description). The average total years of education was 12.7. Almost two thirds of the participants were unemployed (*n* = 170, 63%). The majority (*n* = 213, 80%) of the participants were single, including divorced or widowed with 20% married or living with a partner. Mean premorbid social adjustment was 1.8 (SD 1.6) and academic adjustment 2.4 (SD 1.4).

**Table 1:** Sample description.

**Sociodemographic variables *N* = 269**	**Mean + Standard deviation Number (%)**
Males	161 (60%)
Age	27.2 ± 8.2
Total years of education	12.7 ± 2.6
Employed/Student	99 (36.8%)
Married/Co-inhabitant	55 (20.4%)
Premorbid Social adjustment	1.78 ± 1.6
Premorbid Academic adjustment	2.4 ± 1.4
Immigrant background	78 (29%)
First generation immigrants	37 (13.8%)
Second generation immigrants	11 (4.1%)
Other	30 (11.2%)
Migration after the age of 6	31 (11.5%)
Non-caucasian	50 (18.6%)
**Diagnostic distribution**	
Schizophrenia spectrum disorders	137 (51%)
Bipolar disorders	59 (22%)
Other psychotic disorders	73 (27%)

Of the total sample, 29% (*n* = 78) had immigrant background. Our study included a slightly higher percentage of immigrants than that found in Oslo, Norway in 2008 (25%) (21). Almost 14% were first-generation immigrants with age of migration varying from 0 to 38 years. Thirty-one immigrants migrated after the age of 6 (11.5%). Of these 10 participants migrated from other European countries (32.3%), 13 from Asia including Turkey (42%), 7 from African countries (22.6%), and 1 from South America (3.2%). Because of the wide ethnic background and small samples when stratified, ethnicity was for the statistical analyses dichotomized into Caucasian/non-Caucasian (18/82% for those who migrated before the age of six, and 29/71% for those who migrated after the age of six).

### Duration of Untreated Psychosis

The median DUP was 30 weeks (*IQR* = 126), and the age at onset was 22 years (*IQR* = 10). Bivariate analyses showed significant associations between longer DUP and fewer years of education, lower PAS scores, a diagnosis of schizophrenia spectrum disorder, and younger age of onset (Tables 2, 3). We did not find DUP to be significantly longer in immigrants or those who migrated after the age of six in this sample.

**Table 2:** Comparison of duration of untreated psychosis (DUP) across sociodemographic and clinical characteristics.

***N* = 269**	***M***	***SD***	**Statistics**	***df***	**(95%*CI*)**	***P***
**Gender**
Male (*n =* 161)	1.49	−0.81	*t* = −0.56	267	(−0.26, 0.14)	0.58
Female (*n =* 108)	1.44	−0.82				
**Caucasian/Non–caucasian**
Caucasian (*n =* 219)	1.43	0.82	*t* = 1.67	267	(−0.038, 0.46)	0.095
Non–Caucasian (*n =* 50)	1.64	0.71				
**Immigrant background**
No (*n =* 191)	1.41	0.81	*t* = −1.84	267	(−0.41, 0.01)	0.067
Yes (*n =* 78)	1.61	0.78				
**Migration after the age of six**
Yes (*n =* 31)	1.62	0.86	*t* = −1.14	267	(−0.48, 0.13)	0.254
No (*n =* 238)	1.45	0.81				
**Diagnosis**
Schizophrenia spectrum (*n =* 137)	1.79	0.71	*F* = 65.17^a^	2, 143.42		< 0.001
Bipolar disorder (*n =* 59)	0.71	0.56				
Other (*n =* 73)	1.50	0.76				

a *Welch's F-test reported. PAS = Premorbid Adjustment Scale. DUP is logtransformed*.

**Table 3:** Correlation matrix.

		**1**	**2**	**3**	**4**	**5**	**6**	**7**	**8**	**9**	**10**
1	DUP	1									
2	Gender	0.034	1								
3	Age	0.093	−0.011	1							
4	Age of onset	−0.289^**^	0.033	0.786^**^	1						
5	PAS social	0.290^**^	0.065	−0.020	−0.032	1					
6	PAS academic	0.174^**^	0.120^*^	−0.126^*^	−0.102	0.342^**^	1				
7	Schizophrenia	0.396^**^	0.000	−0.090	−0.185^**^	0.261^**^	0.079	1			
8	Caucasian	−0.102	−0.001	0.026	−0.014	0.088	−0.019	−0.106	1		
9	Migration after age of 6	0.070	0.034	0.142^*^	0.075	−0.067	−0.087	0.075	−0.486^**^	1	
10	Total years of education	−0.232^**^	−0.136^*^	0.307^**^	0.322^**^	−0.148^*^	−0.453^**^	−0.234^**^	0.188^**^	−0.059	1
11	Recognition of psychotic symptoms	−0.169^**^	−0.058	−0.161^**^	−0.118	−0.005	−0.037	−0.077	0.249^**^	−0.239^**^	0.126^*^

** *Correlation is significant at the 0.01 level (2-tailed)*.

* *Correlation is significant at the 0.05 level (2-tailed). Duration of untreated psychosis (DUP) is logtransformed for correlation analysis*.

### Recognition of Psychotic Symptoms

Over half of the participants (61%) were able to recognize psychosis, while 21% attributed it to general mental disorder, and 18% had other explanations (See Table 4). There was a significant difference in DUP between these groups, with the group detecting psychosis having a significantly shorter DUP than the group with other explanations. The group who recognized psychotic symptoms were significantly younger than those who had other explanations. There were however no significant differences in education measured in years. A higher percentage of those who migrated after 6 or were non-Caucasian did not recognize the vignette as psychosis but rather mental illness or had other explanations.

**Table 4:** Group comparison by recognition of psychotic symptoms.

	**1**	**2**	**3**			
	**Psychosis**	**Mental disorder**	**Other explanations**	**Kruskal- Wallis**	**Bonferroni correction**	***P***
	***N =* 165**	***N* = 57**	***N =* 47**	**DF 2**		
DUP	Median 26 (0–1,040)	Median 54 (0–912)	Median 78 (1–1,040)	8.847	1 < 3	0.012
				***F*****–test**		
Age	26.1 ± 7.5	28.3 ± 8.6	29.3 ± 9.8	3.740	1 < 3	0.025
Education (in years)	12.9 ± 2.7	12.1 ± 2.5	12.6 ± 2.6	2.325		0.100
				***X**^**2**^*	**Standardized residual** **>** **2**	
Migration after 6	9 (5.5%)	13 (22.8%)	9 (19.7%)	15.758	1 < 2,3	0.001
Non–caucasian	18 (10.9%)	17 (29.8%)	15 (31.9%)	16.702	1 < 2	0.001

### Migration, Education, and Recognition of Psychotic Symptoms

**Figure 1:**
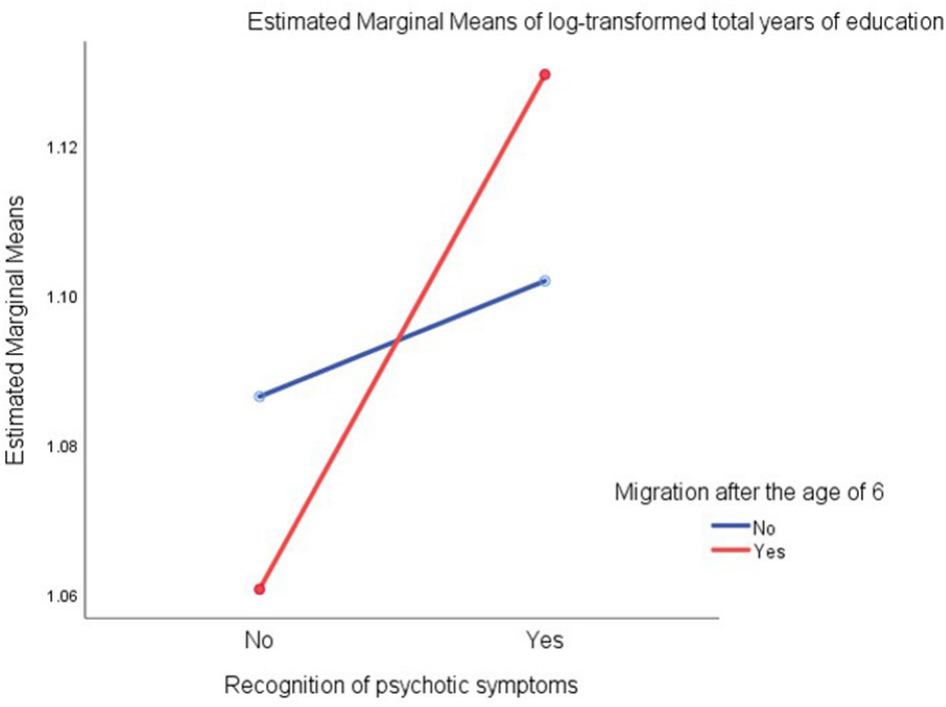
Relationships among total years of education (log-transformed), recongnition of psychotic symptoms, and migration experience after the age of six generated by test of between subjects effects with ANOVA.

The relationship between migration after the age of six, education and recognition of psychotic symptoms was assessed in a two-way ANOVA and can be seen in Figure 1. Those who recognized psychotic symptoms had in average a longer education than those who did not, and this difference was greater in the group who migrated after the age of six. However, this interaction effect did not reach the level of statistical significance (*F* = 2.253, df = 3, *p* = 0.083).

Interaction effects between DUP and recognition of psychotic symptoms and migration after the age of six were also assessed in a two-way ANOVA (*F* = 2.718, *df* = 3, *p* = 0.045). The assumed interaction between recognition of psychotic symptom and migration after the age was not significant (*F* = 0.117, *df* = 1, *p* = 0.73).

### Hierarchical Multiple Regression Analysis of DUP

As can be seen in Table 5, neither migration after the age of six nor total years of education contributed significantly to a prediction model of DUP after including other predictors. However, recognition of psychotic symptoms predicted an additional 2.3% of variance in DUP. The model had a good fit and explained ~26% of the variance in DUP [*F*_(8, 260)_ = 12.55, *p* < 0.001]. The interaction term between recognition of psychotic symptoms and total years of education was put into the model separately and did not contribute significantly toward a better model [Δ*F*_(1, 259)_ = 2.28, *p* = 0.132].

**Table 5:** Hierarchical regression predicting duration of untreated psychosis (DUP).

**Predictors**	**Cumulative**			**Unstandardized**		**Standardized**			**95.0% Confidence Intervals**	
	**Adjusted *R*^**2**^**	**Δ*R*^**2**^**	***ΔF***	***B***	***SE***	**β**	***t***	**Sig**.	**Lower bound**	**Higher bound**
(Constant)				2.949	0.748		3.945	0.000	1.477	4.421
**Block 1**
Gender	−0.003	0.001	0.313	0.018	0.089	0.011	0.201	0.841	−0.157	0.192
**Block 2**
PAS social	0.080	0.089	13.023^**^	0.606	0.184	0.192	3.291	0.001	0.243	0.968
PAS academic				0.229	0.274	0.053	0.836	0.404	−0.310	0.769
**Block 3**
Age at onset	0.233	0.157	27.476^**^	0.451	0.092	0.278	4.886	0.000	0.269	0.632
Schizophrenia				−1.318	0.309	−0.244	−4.262	0.000	−1.927	−0.709
**Block 4**
Migration after age of 6	0.237	0.007	2.343	0.115	0.140	0.045	0.822	0.412	−0.160	0.390
**Block 5**
Total years of education	0.235	0.001	0.301	−0.109	0.607	−0.012	−0.180	0.857	−1.305	1.086
**Block 6**
Recognition of psychotic symptoms	0.256	0.023	8.410^*^	−0.267	0.092	−0.161	−2.900	0.004	−0.449	−0.086

*Overall F_(8, 260)_ = 12.55, p < 0.00*.

** * significant at the 0.01 level*,

* *significant at the 0.05 level. PAS = Premorbid Adjustment. The dependent variable of DUP is logtransformed*.

## Discussion

In our sample of first episode psychosis patients, we found that the effect of MHL was greater than that of length of education or migration history in predicting DUP. It has been postulated that MHL is influenced by an interaction between migration history and years of education, but this interaction was not detected in our sample. We did however find that DUP was bivariately associated with education, level of premorbid function, age at onset, and diagnosis, in line with previous findings (30–33), but that education was not found to be a predictor of DUP when controlling for other variables. Immigrant status *per se* was not a predictor of DUP, but non-Caucasian participants from a variety of ethnic minority groups did have lower recognition of psychosis than other participants.

It has repeatedly been reported that there is no significant association between DUP and ethnicity *per se* (34–37). However, in one large study of over 800 participants it was found that being a first generation immigrant did predict DUP (5). We did not find this in our sample, but it has been found in a larger sample from the same cohort in first generation immigrants who migrated after the age of 6 (6). The reason for this could be that the Nerhus-study included a larger sample (*N* = 462) and thus had better statistical power, however without access to MHL data. Together these findings suggest that ethnic minority status does not predict DUP but having migrated between countries and cultures could have a significant effect.

The risk of schizophrenia and psychosis found in immigrant groups is especially high in migrants from non-European countries who have migrated to European countries or are defined as having black skin color (8, 9). Socioeconomic status does not fully explain increased risk in different context and theories suggest that socio-exclusion may be of importance in the development and outcome of psychosis in underprivileged groups (38). The findings of highest risk in people with black skin color transcends ethnic background as it is found in both black-Caribbean's in England with migration background (39), and African-Americans without an individual history of migration in their lives or the lives of their parents (40). Ethnicity is a multifaceted concept that influences identity and is based on a common history, culture and language, and sometimes religion. Symptom interpretation and illness definitions are influenced by both culture and ethnicity and thus also effect mental health literacy and use of services (41).

Previous studies have suggested an association between ethnicity and MHL (37). Asian patients with psychosis in Norway are found more likely to have alternative explanations about psychosis (20). In the current study we found lower recognition of psychosis in non-Caucasians. These findings indicate that MHL may be influenced by ethnic background, perhaps through cultural variations in illness models. However, in our study, the dichotomy Caucasian/non-Caucasian was highly correlated with migration after the age of six, and we thus could not explore the independent influence of ethnic or cultural background from age at migration. The association between MHL and ethnicity may also be influenced by integration in the residing country, and this should be the subject of future studies.

Even though we found a small correlation between education and MHL, we did not find significant variance in length of education in those who recognized symptoms as psychosis or viewed them as another mental illness or had alternative explanations. Education length did not predict DUP either. There was an interaction between education and MHL among those who migrated after school start, but this did not reach statistical significance. Our study of mental health literacy thus supports previous research in somatic health showing that health literacy is more important in predicting outcome (here DUP) than education and ethnicity (12–14). There are few studies of MHL and education, and they have been conducted in very different contexts. For example a study from Pakistan found low detection of psychosis even in a group viewed as well-educated (42), while higher education was found to be associated with better recognition of depression (psychosis was not assessed) in an Australian sample in tertiary education (43). We have not found studies assessing education and MHL in immigrant groups and can therefore not make any direct comparisons, however our findings suggest that developing separate and specific MHL campaigns for immigrants with both low and high education may be especially beneficial in reducing DUP.

### Limitations

The results of this study must be viewed in the context of certain limitations. This is a cross-sectional study, and our results cannot explain causality between variables. There is also a possible selection bias as the study only included people who could communicate in a Scandinavian language and were legal immigrants, limiting marginalized groups in participation to the study. These are groups suspected of having even less MHL and therefore perhaps higher DUP. Inclusion of marginalized groups is an important endeavor for future studies. Still we had a higher percentage of immigrants in this sample than that found in the same area at the time (21), which suggests both a representative referral of immigrant participants to our study but also that this may be a group presenting more psychotic symptomatology than the majority population. However, the latter cannot be confirmed as this was not an epidemiological study. Possible cultural variations could be of some importance in both MHL and DUP but as this was not the focus of this study it was not systematically assessed. Persons with psychotic disorders receiving treatment in the catchment area were informed of the study by their primary therapist, but we do not have information on those who declined to participate, and this may also have influenced the generalization of our findings. However, there are no alternative treatment practices in the area, reducing recruitment bias.

The relatively high levels of MHL compared to previous studies (16, 44) could be because our participants had themselves experienced psychosis and already received psychoeducation. In the general population Jorm et al. (16) found that 27% could correctly identify schizophrenia after reading a vignette, while Cotton et al. (44) found similar percentages in young people between 12 and 25, with 28% females and 21% males correctly recognizing psychosis. However, these studies are from 1997 and 2006, and MHL in the general public may have changed in the last two decades. We were not able to measure mental health literacy before the onset of psychosis, but of interest are the findings that even after having experienced and been treated for a psychosis and being asked about symptoms and insight for this research project, there are still participants (42%) who do not believe that the vignette of psychotic symptoms is an expression of psychosis. There are few studies of MHL in persons who are already receiving treatment for mental illness, but one such study found that having experienced a problem similar to the MHL vignette is found to be associated with lower MHL scores on the early schizophrenia mental health scale (45). Prospective studies would be able to clarify this matter further.

A final limitation is our sample size, which had it been larger may have strengthened the results. However, as this is the first study of the association between MHL, education, and migration in DUP it gives grounds for further exploration in this area of research.

## Conclusion

MHL, measured as recognition of psychotic symptoms, had a small but significant effect in predicting DUP, independent of years of education and migration history. There was indication that education level was more important to MHL in migrant groups, but this must be assessed in larger populations. This information is important in the customization of MHL campaigns. Interventions that work well for the majority population in industrialized, democratic, and well-educated nations might be ineffective for ethnic minority groups from different cultural backgrounds because of incongruences with illness beliefs, and therefore, a MHL intervention should be built on a more culturally informed framework (46). The knowledge generated by the current study can be used in tailoring interventions more responsive to groups who may not have benefited from Norway's nation-wide efforts to cultivate MHL and to facilitate access to treatment in schools (47, 48).

## Data Availability Statement

The raw data supporting the conclusions of this article will be made available by the authors, without undue reservation.

## Ethics Statement

The studies involving human participants were reviewed and approved by The Regional Committee for Medical Research Ethics (ref # 2009/2484), South-Eastern Norway. The patients/participants provided their written informed consent to participate in this study.

## Author Contributions

NT has written the manuscript and is responsible for the conception and data analyses in cooperation with AO. IM is responsible for project funding and has contributed in writing, analysis, and editing of the manuscript. EB and MN have contributed with insight, scientific discussion, and editing of the manuscript. IM, AO, EB, and MN have all contributed with data collection. All authors contributed to the article and approved the submitted version.

## Conflict of Interest

The authors declare that the research was conducted in the absence of any commercial or financial relationships that could be construed as a potential conflict of interest.

## Publisher's Note

All claims expressed in this article are solely those of the authors and do not necessarily represent those of their affiliated organizations, or those of the publisher, the editors and the reviewers. Any product that may be evaluated in this article, or claim that may be made by its manufacturer, is not guaranteed or endorsed by the publisher.

## Abbreviations

DUP: Duration of untreated psychosis
MHL: Mental health literacy
PAS: Premorbid adjustment scale
PANSS: Positive and Negative syndrome scale.
